# Inverting singlet and triplet excited states using strong light-matter coupling

**DOI:** 10.1126/sciadv.aax4482

**Published:** 2019-12-06

**Authors:** Elad Eizner, Luis A. Martínez-Martínez, Joel Yuen-Zhou, Stéphane Kéna-Cohen

**Affiliations:** 1 Department of Engineering Physics, École Polytechnique de Montréal, Montréal, H3C 3A7 QC, Canada.; 2 Department of Chemistry and Biochemistry, University of California, San Diego, La Jolla, CA 92093, USA.

## Abstract

In organic microcavities, hybrid light-matter states can form with energies that differ from the bare molecular excitation energies by nearly 1 eV. A timely question, given the recent advances in the development of thermally activated delayed fluorescence materials, is whether strong light-matter coupling can be used to invert the ordering of singlet and triplet states and, in addition, enhance reverse intersystem crossing (RISC) rates. Here, we demonstrate a complete inversion of the singlet lower polariton and triplet excited states. We also unambiguously measure the RISC rate in strongly coupled organic microcavities and find that, regardless of the large energy level shifts, it is unchanged compared to films of the bare molecules. This observation is a consequence of slow RISC to the lower polariton due to the delocalized nature of the state across many molecules and an inability to compete with RISC to the dark exciton reservoir.

## Teaser

Molecules sandwiched in an optical cavity can form hybrid light-matter states at energies below the dark spin triplet state.

## INTRODUCTION

In the molecular orbital picture, when an electron is promoted from the highest occupied molecular orbital (HOMO) to the lowest unoccupied molecular orbital (LUMO), it can form a bound electron-hole pair, termed an exciton, with either singlet (total spin *S* = 0) or triplet (*S* = 1) character. Under electrical excitation of organic thin films, where uncorrelated charges are injected, spin statistics dictate that when oppositely charged carriers meet on a single molecule, they will form triplet excitons three times more often than singlet excitons (*1*). In contrast, under optical excitation, singlet excitons are preferentially formed because triplet exciton formation is spin forbidden from the singlet ground state. However, spin mixing due to weak spin-orbit coupling or the hyperfine interaction can lead to a subsequent transition from the singlet to the optically dark triplet state, a process known as intersystem crossing (ISC). One important characteristic of triplets is that their energy is almost always below that of the singlet because of the positive value of the Coulomb exchange integral. The singlet-triplet gap, Δ*E_ST_*, is typically on the order of ~0.5 to 1 eV, which makes thermally activated reverse ISC (RISC) from the triplet to the singlet very inefficient compared to typical nonradiative decay rates (*2*). Triplet excitons in organic molecules are thus long lived with very low radiative efficiencies for transitions to the ground state, a process known as phosphorescence. This has been a source of important challenges for the development of efficient organic light-emitting diodes (OLEDs) and organic lasers.

A common approach to overcome losses due to triplets is the use of molecules containing heavy noble metals that greatly increase phosphorescence rates because of spin-orbit coupling (*1*). This approach has been very successful, leading to 100% internal quantum efficiency in OLEDs. However, organometallic complexes have some disadvantages such as their high cost due to the use of metals such as platinum and iridium, limited photostability for blue emitters, and possible toxicity (*3*, *4*). In recent years, there has been a tremendous effort to develop purely organic emitters that can harvest triplets by converting them back to singlets through processes such as triplet-triplet annihilation or thermally activated delayed fluorescence (TADF) (*3*–*5*). In TADF materials, the singlet-triplet energy gap is minimized (<0.1 eV), typically through donor-acceptor molecular design, which reduces the spatial overlap between HOMO and LUMO wave functions. This allows for thermally activated RISC of triplets to the singlet state, followed by efficient fluorescence. It has been shown that this approach can lead to the conversion of nearly all triplets into emissive singlet states (*3*). One disadvantage of this approach is that RISC rates are typically slow (with a rate constant of ~10^6^ s^−1^), which can lead to large buildups of triplets at high excitation densities and subsequent losses due to triplet-triplet and triplet-charge quenching.

It has been known for some time that optical microcavities can be used to modify the energy of singlet excitons through the formation of system eigenstates called polaritons. An outstanding and timely question is, To what extent can these modified energetics affect triplet dynamics? (*6*–*8*). In the ultimate case where singlets and triplets are inverted energetically, can RISC be made very efficient to obtain high TADF efficiencies for a broad range of materials and avoid the buildup of large triplet populations?

Polaritons are light-matter eigenstates that form when singlet electronic transitions are strongly coupled to the vacuum electromagnetic field in an optical cavity. This occurs when the light-matter interaction rate is faster than the photon and electronic decay rates in the system. In the past decade, organic polaritons have proved to be a remarkable platform for the demonstration of fascinating nonlinear quantum phenomena at room temperature (*9*–*11*), the development of novel optoelectronic device applications (*12*–*15*), and modification of chemical reactions (*16*–*21*). Polariton states are separated into two types of dispersive modes, each on different sides of the electronic transition, called lower polaritons (LPs) and upper polaritons (UPs). The minimal separation between LP and UP states occurs when the cavity photon energy is resonant with the electronic transition and is termed the vacuum Rabi energy (Ω). As a result, the energetic separation between LPs and the triplet is smaller than the separation between triplets and the peak singlet absorption. For a large number of molecules, *N*, the Rabi energy $\Omega \;\propto \;\sqrt{N/V}$, where *V* is the cavity mode volume. Because of the large number of molecules in a typical cavity as compared to the number of photonic modes supported by the cavity, *N*_ph_, strong coupling leads to the formation of *N–N*_ph_ dark states at the same energy as the singlet transition, commonly referred to as the exciton reservoir. Even in the presence of disorder, these states are largely molecular (rather than photonic) in character and, because of their large number, have been shown to dominate the dynamics of polariton relaxation (*20*, *22*, *23*).

Experiments on triplet dynamics where the singlet transition is strongly coupled to a cavity mode were performed as early as 2007. In that case, it was found that resonantly excited polaritons could intersystem-cross to the triplet but that dark states dominated the overall dynamics and no substantial changes in rates were observed (*6*). Very recently, experiments with much larger Rabi energies observed a modest reduction of phosphorescence decay rate in strongly coupled optical microcavities, which was attributed to polariton-enhanced RISC (*7*). Phosphorescence rates, however, are sensitive to any changes in the optical environment, such as the presence of mirrors, so it is unclear to which extent changes were due to radiative effects or to polaritonic effects.

Here, we demonstrate the formation of polaritons in a material showing TADF, where we are able to convincingly invert the singlet polariton and the triplet-state energies. We directly measure triplet dynamics via the RISC rate, which is independent of any modifications in the optical density of states from the presence of the microcavity. We show that although RISC can be understood as a thermally activated Marcus process, reordering of the states leads to negligible changes in the overall dynamics of the system. In essence, because ISC is a single-molecule process, rate constants describing relaxation from the triplet to the delocalized singlet polariton scale as 1/*N*_eff_, where *N*_eff_ is the effective number of molecules coupled to each cavity mode. Given that *N*_eff_ ~ 10^6^ in uniformly filled planar cavities, any contribution from the reordering is strongly suppressed. Last, we want to highlight the importance of doing these experiments in vacuum to avoid artificial modifications of triplet lifetimes due to quenching from triplet oxygen (see fig. S1) and in the linear excitation regime. In control experiments, we find that simple encapsulation from molecular oxygen due to the presence of microcavity mirrors can lead to large changes in rates.

## RESULTS

**Fig. 1:**
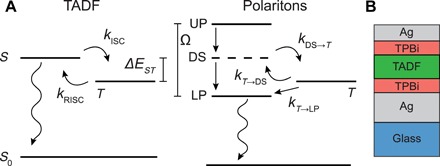
Photophysical kinetic diagrams and microcavity structure. (**A**) Electronic energy levels and rate constants for the TADF material and polaritons. (**B**) Microcavity structure consisting of a Ag bottom mirror (100 nm), a Ag top mirror (30 nm), TBPi buffer layers (10 nm each), and a TADF layer consisting of either neat 3DPA3CN with a thickness of 70 nm (MC Neat) or codeposited TPBi-3DPA3CN (55 to 45% by volume) with a thickness of 64 nm (MC 1) or 81 nm (MC 2).

Figure 1A shows a simplified diagram of the electronic energy levels and rate constants for TADF molecules and for polaritons in this material. When TADF molecules are optically excited, charge transfer singlets (^1^CT) are rapidly formed (*5*). Radiative decay can then occur either directly as prompt fluorescence or through ISC followed by RISC in a process known as delayed fluorescence. The thermally activated RISC rate constant shows a Boltzmann dependence (*3*, *4*)

$$k_{\text{RISC}}=A\;\text{exp}(-\frac{E_{a}}{k_{B}T})$$ (1)

where *k*_B_ is the Boltzmann factor, *T* is the temperature, and *E*_a_ is the activation energy. In principle, the activation energy depends both on the singlet-triplet energy gap and the singlet-triplet reorganization energy, λ*_T_*. In TADF molecules that show efficient RISC, the activation energy is expected to be similar to the singlet-triplet energy gap (*3*, *4*). As shown in recent works (*24*, *25*), the mechanism for ISC and RISC in TADF materials involves strong vibronic coupling between local triplet states (^3^LE) and the triplet charge transfer states (^3^CT), followed by spin-orbit coupling to ^1^CT states. For simplicity, we will use the notation *S* and *T* in Figs. 1 and 5 to describe the states of singlet and triplet character that undergo ISC.

In a polariton setup, optical excitation at energies above that of the singlet exciton, such as at the UP or at even higher energies, leads to rapid relaxation to the dark states (*22*, *23*, *26*). As a result, light emission is mostly due to relaxation from the dark states to the LPs, followed by the radiative decay of LPs. The latter process occurs on a scale given by the cavity lifetime (<10 fs), which can be considered as instantaneous compared to the other time scales in the system. This combination leads to prompt radiative decay upon optical excitation. In materials that show TADF, however, dark states can also undergo ISC, which through RISC can ultimately populate the LP. This process leads to delayed fluorescence due to the slow rate typical of RISC. As shown in Fig. 1A, the delayed fluorescence pathway will involve ISC to the triplet, followed by triplet to dark state RISC with a rate constant of *k*_*T* → DS_ and triplet to LP RISC with a rate constant of *k*_*T* → LP_. The first rate constant is essentially unchanged from that in the bare system, while the second rate constant is unique to the microcavity. If it could be made faster than *k*_*T* → DS_, then it would provide the means for accelerating RISC compared to the case of the bare molecule.

**Fig. 2:**
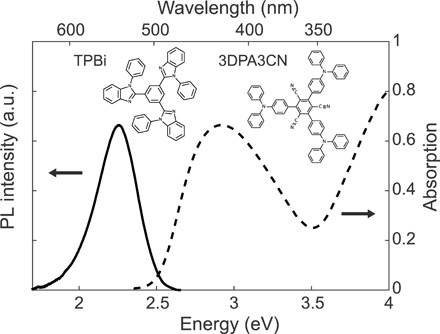
Chemical structure and optical properties of 3DPA3CN. Absorption (dashed line) and PL (solid line) spectra of 3DPA3CN neat film. Inset shows the chemical structure of 3DPA3CN and TPBi (used as the host in mixed samples). a.u., arbitrary units.

The molecule that we chose for this study is a trigonal donor-acceptor of 1,3,5-tris(4-(diphenylamino)phenyl)-2,4,6-tricyanobenzene (3DPA3CN) (*27*). This molecule has been shown to have a singlet-triplet energy gap of Δ*E_ST_* = 0.1 eV and slow (*k*_RISC_~2 × 10^3^ s^−1^) but efficient thermally activated RISC. In addition, 3DPA3CN shows no phosphorescence even at low temperature. The absorption and photoluminescence (PL) of a neat film of 3DPA3CN is shown in Fig. 2. The absorption has a peak at 425 nm (2.92 eV) due to a CT^1^ excited state, and the PL is Stokes-shifted by 0.66 eV with a peak at 549 nm (2.26 eV). PL quantum efficiency (PLQE) was measured using an integrated sphere with vacuum-packed samples (see Materials and Methods). The neat film showed a PLQE Φ = 86 ± 3%, and when codeposited with 2,2′,2″-(1,3,5-benzinetriyl)-tris(1-phenyl-1-*H*-benzimidazole) (TPBi) to reduce aggregation-induced quenching in the film, the PLQE reaches Φ = 98 ± 3% (see Table 1). Three microcavities were fabricated (see the structure in Fig. 1B), consisting of either neat 3DPA3CN with a thickness of 70 nm (sample MC Neat) or codeposited TPBi-3DPA3CN (55 to 45% by volume) with thicknesses of either 64 nm (MC 1) or 81 nm (MC 2). The active layer was sandwiched between two 10-nm TPBi buffer layers and 100-nm (30 nm) Ag bottom (top) mirrors. The buffer layers were used to minimize any direct quenching from the metal. In addition, control samples consisting of the active and buffer layers without any mirrors were fabricated during the same deposition run by masking the metal deposition.

**Table 1:** Photophysical data of the microcavities and control samples. MC 1 (Ω = 0.45 eV, Δ = −0.36 eV), MC 2 (Ω = 0.5 eV, Δ = −0.49 eV), and MC Neat (Ω = 0.87 eV, Δ = −0.58 eV). When not specified, measurements were performed at room temperature. Notes: (i) *k*_ISC_ for the microcavities were taken from the control values. (ii) The PLQEs for 100 and 200 K were estimated from the measured yields: Φ_PF_(200 K)/Φ_PF_(300 K) = 1.02, Φ_PF_(100 K)/Φ_PF_(300 K) = 1.05 for the control, and Φ_PF_(100 K)/Φ_PF_(300 K) = 1.01 for MC 2. (iii) The uncertainties of *k*_d_ and *k*_p_ were estimated from the fit’s SD errors. For the PLQEs, a constant 3% uncertainty error was assumed. (iv) Even for long integration times, the delayed signal at 100 K is too weak to reliably extract the delayed lifetime. (v) From the two data points of the RISC rate constants at 200 and 300 K using Eq. 1, we obtain activation energy, Δ*E*_a_ = 60 meV.

**Sample**	***k*_p_ × 10^8^ [s^−1^]**	***k*_d_ × 10^3^ [s^−1^]**	**Φ_d_/Φ_p_**	**Φ**	***k*_ISC_ × 10^7^ [s^−1^]**	***k*_RISC_ × 10^3^ [s^−1^]**
MC 1	2.2 ± 0.1	2.5 ± 0.3	4.0%	7.5 ± 3%	`1.4 ± 0.6	1.5 ± 0.6
Control 1	1.8 ± 0.1	2.6 ± 0.2	6.3%	98 ± 3%	1.4 ± 0.6	2.1 ± 0.8
MC 2	2.2 ± 0.1	3.2 ± 0.2	4.5%	32 ± 3%	1.4 ± 0.4	2.3 ± 0.7
Control 2	1.4 ± 0.1	2.8 ± 0.3	5.4%	95 ± 3%	1.4 ± 0.4	1.5 ± 0.5
MC 2 (200 K)	2.2 ± 0.1	3.4 ± 0.1	1.2%	31 ± 3%	1.3 ± 0.5	0.7 ± 0.3
Control 2 (200 K)	1.6 ± 0.1	3.3 ± 0.3	1.2%	93 ± 3%	1.3 ± 0.5	0.5 ± 0.2
MC 2 (100 K)	2.3 ± 0.1	–	0.2%	32 ± 3%	0.9 ± 0.5	–
Control 2 (100 K)	1.7 ± 0.1	–	0.3%	95 ± 3%	0.9 ± 0.5	–
MC Neat	3.0 ± 0.1	10.9 ± 1.8	0.7%	20 ± 3%	2.8 ± 0.6	0.8 ± 0.2
Control Neat	1.9 ± 0.1	11.3 ± 0.9	0.7%	86 ± 3%	2.8 ± 0.6	0.6 ± 0.1

**Fig. 3:**
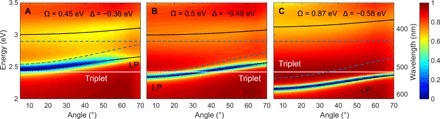
Angle-resolved reflectivity. Measured using TE polarized light for (**A**) MC 1, (**B**) MC 2, and (**C**) MC Neat (as defined in the text). The solid black lines show the least-square fit to the Hopfield Hamiltonian (see Materials and Methods). The dashed blue lines are the energies corresponding to the uncoupled photonic mode and the exciton singlet absorption. The solid white lines are the triplet energies, which are 0.1 eV (*27*) lower in energy than the bottom of the singlet band.

To extract the polariton dispersion relation, we measured the angle-resolved reflectivity of the fabricated microcavities, which is shown for transverse electric (TE) light polarization in Fig. 3 (A to C, respectively). For each incident angle, we observe two minima corresponding to the excitation of the LP and UP states. The dashed blue lines show the uncoupled singlet exciton absorption peak (*E*_x_) and the cavity photon energies. The black lines show a least squares fit of the minima to the Hopfield Hamiltonian (see Materials and Methods). The triplet energy is shown as a solid white line at 2.41 eV, which corresponds to an energy of 0.1 eV lower than the bottom of the singlet band as previously reported (*27*). This value is also consistent with our temperature-dependent measurements reported below. The energy of the bottom of the singlet, which corresponds to the 0-0 transition, was taken to be at 2.51 eV from the crossing energy between the PL and absorption spectra (see Fig. 2). This should be contrasted with the absorption maximum, which corresponds to the vibronic transition with the strongest Franck-Condon overlap. From the fits to the Hopfield Hamiltonian, for MC 1 (Fig. 3A), we obtain a Rabi energy of Ω = 0.45 ± 0.02 eV with detuning of Δ = *E*_c_ − *E*_x_ = −0.36 ± 0.02 eV for the TE mode, where *E*_c_ is the cavity photon energy at normal incidence, and Ω = 0.40 ± 0.01 eV for the transverse magnetic (TM) mode (see table S1) (*28*). As can be seen from Fig. 3A, the LP energies at all incident angles are higher than the triplet energy for MC 1. For a thicker TADF layer, i.e., MC 2 (Fig. 3B), we obtain a Rabi energy of Ω = 0.50 ± 0.02 eV with detuning of Δ = −0.49 ± 0.03 eV for the TE mode and Ω = 0.42 ± 0.02 eV for the TM mode. As can be seen from Fig. 3B, the LP energies for incident angles above 35° are higher than triplet energy, but remarkably for incident angles below 35°, the LP energies are lower than triplet energy. Last, for a neat TADF layer (MC Neat), we obtain a Rabi energy of Ω = 0.87 ± 0.01 eV with detuning of Δ = −0.58 ± 0.02 eV for the TE mode and Ω = 0.87 ± 0.02 eV for the TM mode. This Rabi energy corresponds to 30% of the uncoupled singlet exciton energy (Ω/*E*_x_), entering the realm of ultrastrong light-matter coupling (*29*). As can be seen from Fig. 3C, the LP energies for all incident angles are lower than the triplet energy, completely inverting the singlet-triplet ordering of the system for all of the measured angles.

Notably, the measured PLQEs for the microcavities were found to be systematically lower than those of the control samples (see Table 1). The lower quantum efficiencies can be attributed to slow relaxation from the dark states to the LP, relaxation of dark states to TM metal-insulator-metal plasmonic modes with wave vectors beyond the light line, and ohmic losses of the LP mode. As can be seen from the angle-resolved PL spectra in fig. S2, the PL of all microcavities is dominated by LP emission. The lowest PLQE, Φ = 7.5 ± 3%, was measured for MC 1 in which a large portion of the LP states have energies that are higher than the energy of bottom of singlet dark states. The inability to populate these LP states can be seen in the spectra as a sharp drop in PL intensity for any wavelengths below the 0-0 transition at ~490 nm (see fig. S2A). Other microcavities, which had energies below the dark states, do not show this effect and had PLQEs of Φ = 32 ± 3% and Φ = 20 ± 3% for MC 2 and MC Neat, respectively.

A direct way to examine the effect of triplet transitions to the LP is by measuring the delayed fluorescence and comparing the RISC rate constants of the microcavities to the control samples. The RISC rate constant can be calculated from experimental observables using the following equation (*3*, *30*, *31*)

$$k_{\text{RISC}}=\frac{k_{p}k_{d}\Phi_{d}}{k_{\text{ISC}}\Phi_{p}}$$ (2)

where *k*_p_ and *k*_d_ are the prompt and delayed fluorescence rate constants, respectively, Φ_p_ and Φ_d_ are the PLQYs of the prompt and delayed components (Φ = Φ*_p_* + Φ*_d_*), and *k*_ISC_ is the ISC rate constant. The latter was estimated from the control sample using *k*_ISC_ ≈ *k*_p_(1 − Φ_p_), assuming that *k*_ISC_ is the dominant nonradiative decay pathway for the singlet.

**Fig. 4:**
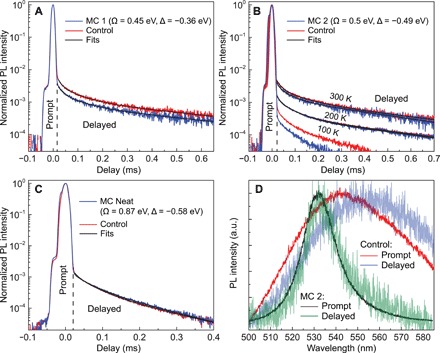
Transient delayed PL characteristics. Transient PL decays for the LP (blue line) and control film singlet (red line) at a collection angle of 0°. (**A**) MC 1, (**B**) MC 2, and (**C**) MC Neat (as defined in the text). The black lines are multiexponential fits to the delayed signal decay. (**D**) Delayed and prompt PL spectra acquired by an integration over the prompt and delayed parts of the decay for MC 2 and control at room temperature.

To obtain RISC rate constants, transient PL was measured for all of the samples (see Materials and Methods). All measurements were performed under vacuum and at laser powers that ensured that the dynamics stayed in the linear regime (see figs. S1 and S3). The normalized time-dependent PL intensities are shown in Fig. 4 (A to C). The large peak close to 0 ms corresponds to the prompt component, followed by the slow delayed fluorescence due to RISC and re-emission. Polariton transients were measured at normal incidence at the corresponding LP energy. To measure the delayed luminescence rate constants, *k*_d_, mean lifetimes were calculated from a multiexponential fit to the delayed decay luminescence data (see Materials and Methods); the values are shown in Table 1. The delayed and prompt efficiencies, Φ_d_/Φ_p_, can be calculated by integrating over the PL intensities of the respective delayed and prompt components (*3*). As can be seen in Table 1, only slight changes were found between the microcavities and the control samples. For the neat samples, *k*_d_ is higher and Φ_d_/Φ_p_ is lower compared to TPBi mixed samples. This is due to concentration quenching at the higher molecular concentration, which is known to have a detrimental effect on triplet lifetimes (*3*).

The prompt PL decays are shown in fig. S4, and the prompt rate constants *k*_d_ are summarized in Table 1. The prompt lifetimes were found to be τ_p_~5 ns, four orders of magnitude shorter than the delayed lifetimes. We find a slightly faster decay rate constant in the microcavities compared to the controls (up to a factor of ~1.6). This is principally attributed to a modification of the local density of optical states inside the microcavities. However, relaxation from the dark states to the LP can also potentially reduce the prompt lifetime if it is fast enough to compete with the radiative decay rate constant. Last, the RISC rate constants can be calculated using Eq. 2. As can be seen in Table 1, no significant changes are found for any microcavity compared to the corresponding controls.

In addition to room temperature studies, we performed transient PL measurements at lower temperatures as shown in Fig. 4B. By reducing the temperature, we increase the effective activation barrier for RISC, Δ*E*_a_/*k*_B_*T*, making the process less efficient. In this case, the rate constant *k*_*T* → LP_ can compete more effectively with *k*_*T* → DS_ and the overall dynamics become more sensitive to the modified energetics in the presence of strong coupling. As can be seen in Fig. 4B and the values in Table 1, at lower temperatures, Φ_d_/Φ_p_ and *k*_RISC_ decrease significantly. Nevertheless, we did not observe substantial modifications in lifetimes or efficiencies in the microcavity compared to the control.

Figure 4D shows the spectra of delayed and prompt components of MC 2 and the control sample. As was observed previously in some TADFs, the delayed spectra of the control are red-shifted compared to the prompt spectra (*3*, *31*). The spectral shift was suggested to occur because of the effect of the triplet excited state on the nuclear configuration of the singlet excited state after RISC. Figure 4D also shows that LPs are the source of the delayed signal in microcavity measurements.

## DISCUSSION

**Fig. 5:**
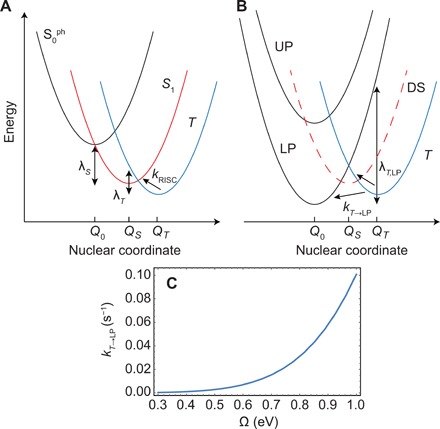
Nuclear potential energy surfaces and population dynamics in the weak and strong coupling regimes. (**A**) In the weak coupling regime, the population accumulated in the triplet electronic manifold needs to overcome a thermal energy barrier to reach the fluorescent singlet electronic state *S*_1_. The population in *S*_1_ needs to further surpass a thermal energy barrier to emit a photon, which corresponds to the state of the system $S_{0}^{\text{ph}}$, the electronic ground state accompanied by a microcavity photon resonant with the vertical singlet transition. (**B**) Upon strong coupling, the energy barriers are different for the dark states and the polariton ones. Transfer from *T* to the LP features a smaller energy barrier compared to the case in (A). However, delocalization of the LP across *N*_eff_ ≈ 10^6^ molecules markedly dilutes the electrostatic coupling between a triplet and a given molecule. This leads to very slow polariton RISC that cannot compete with RISC to the dark states (DS), which occurs at the rate in the bare molecule. This is shown in (**C**), where we calculate polariton RISC rate constants for *N*_eff_ = 4 × 10^6^ and the range of Rabi splittings Ω explored in our experiments (we assume a detuning of Δ = −0.58 eV as in the MC Neat sample at *q* = 0); the computed rate constants are significantly lower than the bare RISC rate constant *k*_RISC_~10^3^ s^−1^.

The above results seem to indicate a negligible influence of the LP mode on the conversion of dark triplet excitons into luminescent ones. To explain this observation, we rely on a theoretical model based on the variational polaron transformation that accounts for the emergent chemical dynamics upon strong coupling (*20*, *32*). The relevant excited states of the molecule-cavity system are shown in Fig. 5 and correspond to $S_{0}^{\text{ph}}$, *S*_1_, and *T*. Here, $S_{0}^{\text{ph}}$is the electronic ground state of the molecule with one photon in the cavity, while *S*_1_ and *T* are the electronically excited singlet and triplet states, respectively, with no photons in the cavity. The relative alignment of the nuclear potential energy surfaces are determined by λ*_S_* = 0.33 eV, the reorganization energy of the singlet $S_{0}^{\text{ph}}-S_{1}$ transition, and λ*_T_* = 0.1 eV, the reorganization energy of the *S*_1_-*T* transition (see Fig. 5). Last, electronic coupling between *S*_1_ and *T* is mediated by the spin-orbit coupling matrix element, *V_ST_*. Given that *V_ST_* is unknown for this material and that an approximate value is sufficient for our purposes, we take *V_ST_* = 2 × 10^−2^ meV, a value characteristic of TADF molecules (*33*, *34*). For 3DPA3CN, λ*_T_* was estimated from Δ*E_st_* and an Arrhenius plot of the RISC rate constant as a function of temperature (*27*). Population transfer between the triplet and singlet state of the bare molecule can be described in terms of a Marcus rate equation, with rate constant *k*_*T* → LP_, if we assume that the transfer rate between singlet and triplet states is mediated by low-frequency modes (which seems to be the case for this particular molecule given the absence of vibronic progressions in the absorption spectrum) that are sufficiently fast compared to the spin-orbit coupling time scale. Theoretical analysis shows that in the strong coupling regime, transfer from the triplet state to the dark singlet states (DS in the figure) also follows an effective Marcus description with rate constant *k*_*T* → DS_ (see Materials and Methods) (*32*).

To find the activation energies of the Marcus description, we first note that the Rabi frequency is larger than the highest frequency vibrational modes coupled to the exciton states. Under these circumstances, the exchange of energy between photon and singlet exciton states is much faster than the coupling of the latter to the vibrational bath. In this case, polaron decoupling ensues and the LP and UP nuclear configurations remain the same as that of the singlet ground state (*19*, *35*), as shown in Fig. 5B. This nuclear rearrangement results in a significantly increased reorganization energy λ_*T*, LP_ for the polariton RISC process when compared to λ*_T_*, with a concomitant increase in the activation energy. This activation energy can, in principle, be suppressed by further increasing the Rabi splitting to decrease the energy of the LP with increased Rabi splitting and yield an exponential increase in *k*_T → LP_. The molecular parameters and light-matter couplings of our experiment correspond to the normal regime of Marcus theory (larger Rabi splittings would be needed to access the inverted regime; see Fig. 5B). Even if the activation energy for transfer to the LP is fully suppressed, the maximum rate constant is bounded at light-matter resonance by $k_{T\to \text{LP}}\leq \frac{{|V_{\mathrm{ST}}|}^{2}}{2\hbar N_{\text{eff}}}\sqrt{\frac{\pi }{\lambda_{T,\text{LP}}k_{B}T}}$, where *N*_eff_ = *N*/*N*_ph_ is the effective number of molecules coupled per cavity mode and ℏ = *h*/2π, with *h* being Planck’s constant. The factor of $\frac{1}{2N_{\text{eff}}}$ in this upper bound arises from the delocalization of the polariton across *N*_eff_ molecules: Half of the polariton has molecular character, and out of this fraction, only $\frac{1}{N_{\text{eff}}}$ corresponds to a singlet that can undergo RISC with a given triplet because spin-orbit interactions are local. If we take *N*_eff_ = 4 × 10^6^ (see Materials and Methods) for the MC Neat sample (*36*), we obtain a room temperature *k*_*T* → LP_ ≤ 0.1 s^−1^ (see Fig. 5C). This value can hardly compete with RISC to the dark states, which occur with a rate constant, *k*_*T* → DS_ = *k*_RISC_, identical to that in the bare molecule.

## CONCLUSION

We have conclusively shown that the RISC rate in a TADF material remains invariant under the strong light-matter regime even under energy inversion of the LP with respect to the triplet energy. Since the RISC process is thermally activated, the corresponding activation barrier to the LP can, in principle, be decreased by increasing the Rabi splitting. However, the large ratio *N*_eff_ between the density of states of the dark triplet states and the polaritonic ones renders an improvement over the rate in the bare molecule unfeasible under normal conditions. This conclusion is independent of the strength of spin-orbit coupling within the material in question (*32*).

We propose two possible avenues to observe polariton-assisted harvesting of triplet excitons based on these observations. First, molecules in which the ISC process is in the so-called inverted Marcus regime are the best candidates to benefit from the formation of polaritons. In this case, the corresponding RISC energy barrier of the bare material will be significantly above Δ*E_st_*, but upon strong coupling within a microcavity, the energy barrier between the triplet states and the LP will decrease and can enter the normal regime. As a result, the cavity-assisted transition from the inverted, with a high RISC energy barrier, to the normal Marcus regime, with a much smaller energy barrier, will result in an exponential increase in the RISC rate constant much larger than that, which is possible in the normal Marcus regime. This exponential gain may be larger than *N*_eff_ in extreme cases. Second, polaritonic states where *N*_eff_ ≈ 100 or less due to extreme mode volume confinement such as in plasmonic cavities (*37*–*39*) can allow for large changes in dynamics. Assuming similar Rabi energies in 3DPA3CN, e.g., this would lead to *k*_*T* → LP_~10^4^ s^−1^, which surpasses the bare RISC rate constant. Last, note that a slow rate constant for the transfer of population from the triplet manifold to the LP may still give rise to an enhancement of the delayed fluorescence yield. This occurs in cases where the equilibrium between singlets and triplets is formed much faster than the rate of singlet fluorescence, such as in singlet fission or organometallic complexes (*8*). Future work will focus on exploring these avenues.

## MATERIALS AND METHODS

### Sample preparation

The devices were fabricated on glass substrates using thermal evaporation at a base pressure of <10^–7^ torr (EvoVac, Angstrom Engineering). Before the deposition of the films, the substrates were cleaned and exposed to an ultraviolet (UV)–ozone treatment. 3DPA3CN was purchased and used as received from Lumtec.

### Characterization

The refractive index and the layer thicknesses were obtained using ellipsometry (J. A. Woollam Co., RC2 D + NIR). Angle-resolved reflectivity measurements were performed using a photospectrometer (Carry 7000). The angle-resolved PL measurements were performed using a fiber-coupled spectrometer (Ocean Optics Flame) with a 405-nm diode laser excitation (Thorlabs, CPS405). Quantum efficiency measurements were performed using an integrated sphere (Labsphere) on samples that were vacuumed in a transparent plastic to avoid exposure to oxygen.

Transient PL characteristics were measured under vacuum (<2 × 10^−4^ torr) using a streak camera (Hamamatsu, C10910) coupled to a spectrometer (Princeton Instruments, SP-2300). The PL signal was collected as 0° using an optical fiber. For delayed lifetime measurements, the samples were excited by a Nd:YAG laser and optical parametric oscillator (440 nm, 10-Hz repetition rate, and 8-ns pulse duration). Typical pump fluences were 10 to 50 μJ/cm^2^. For each sample, measurements were performed at several powers to ensure that the data were taken in a range where no nonlinear (e.g., bimolecular) processes occur. For prompt lifetime measurements, the samples were excited by a supercontinuum laser (Fianium WhiteLaser UV; 0.1-MHz repetition rate, ~50-ps pulse duration, and 405 nm using optical filters). The mean lifetimes were calculated as $\tau_{m}=\frac{\sum_{i}a_{i}\tau_{i}^{2}}{\sum_{i}a_{i}\tau_{i}}$ from multiexponential fits to the data (two terms for the prompt lifetime and three for the delayed), where *a_i_* and τ*_i_* are each exponent weight and lifetime, respectively. The ratios, Φ_d_/Φ_p_, were obtained using integration over the PL decays of delayed and prompt components. Low-temperature measurements were conducted in vacuum using a closed-cycle optical cryostat.

### Dispersion relation fitting

The experimental Rabi splitting and the detuning were extracted for TE or TM mod es by performing a least-square fit of the reflectivity dips to the following linear equation (*28*, *29*)

$$\left(\begin{array}{cccc} E_{\text{ph}}(q)+2D & \Omega /2 & 2D & \Omega /2\\ \Omega /2 & E_{x} & \Omega /2 & 0\\ -2D & -\Omega /2 & -E_{\text{ph}}(q)+2D & -\Omega /2\\ -\Omega /2 & 0 & -\Omega /2 & -E_{x} \end{array})(\begin{array}{c} w_{j,q}\\ x_{j,q}\\ y_{j,q}\\ z_{j,q} \end{array})=E_{j,q}(\begin{array}{c} w_{j,q}\\ x_{j,q}\\ y_{j,q}\\ z_{j,q} \end{array}\right)$$ (3)

Equation 3 is a solution of the Hopfield Hamiltonian, and the eigenvalues correspond to the polariton energies ±*E*_LP, *q*_ and ±*E*_UP, *q*_. The in-plane wave vector is *q*, *j* ∈ {LP, UP} and *D* = Ω^2^/4*E*_x_. We used a photonic cavity dispersion with an effective refractive index *N*_eff_,

$$E_{\text{ph}}(q)=\sqrt{{\left(\frac{\mathrm{hc}q}{N_{\text{eff}}}\right)}^{2}}+E_{c}^{2}$$ (4)

with $q=\frac{\omega }{c}\text{sin}\;\theta$, where θ is the angle of incidence.

### Rate constant calculations

We used a variational polaron transformation of the Hamiltonian (describing electronic states, photonic mode, and vibrational degrees of freedom) to account for the dissipative dynamics that emerge under strong light-matter coupling. This approach introduces an optimal energy partitioning between a vibrationally dressed polaritonic system (featuring renormalized energies of the triplet, singlet, and photonic states, as well as their couplings) and a small perturbation that contains residual couplings mediated by the vibrational bath (*32*). The latter perturbation is dealt with in the Markovian approximation using standard open-quantum systems techniques. For the parameters λ*_S_*, λ*_T_*, and *V_ST_* assumed for 3DPA3CN, the rate constant between any triplet and the LP in the strong coupling regime is well described by a Marcus equation of the form

kT→LP=∣xj,q=0∣2Neff×∣VST∣2ℏπλT,LPkBTexp[−(ΔEST−Ω2+λT,LP)24λT,LPkBT] (5)

where $\lambda_{T,\text{LP}}={\left(\sqrt{\lambda_{S}}+\sqrt{\lambda_{T}}\right)}^{2}$ is the reorganization energy between the triplet and the (polaron decoupled) LP state. We also remind the reader that the reorganization energy between two parabolic surfaces A and B of the same frequency is independent of the direction of transfer (A → B or B → A) or the difference between energy minima but only dependent on their relative displacement from one another. The spin-orbit coupling *V_ST_* is the microcavity-free one, which is not changed upon microcavity confinement because of the negligible effect of the electric field on the angular momentum of the electronic excited states. Notice that the rate constant above contains a prefactor $\frac{{|x_{j,q=0}|}^{2}}{N_{\text{eff}}}$, which equals the probability of finding the singlet in the LP state, which can accept population from a given triplet. We estimate that *N*_eff_ is based on the ratio of the molecular density of states to the cavity photon counterpart (*36*, *40*). The latter is given by $\rho_{\text{ph}}=\frac{{n_{\text{eff}}}^{2}}{4\pi }[{E_{\text{ph}}}^{2}(q_{\text{cut}})-{E_{c}}^{2}]$, where *E*_ph_(*q*_cut_) is the energy cutoff that we chose on the basis of the range of angles, which exhibit the largest PL signals for MC Neat (see fig. S2). By selecting θ = 60^0^ for the latter sample, we have *E*_ph_(*q*_cut_) = 2.6 eV and *E*_c_ = 2.3 eV, which yields ρ_ph_ = 12 μm^−2^. The density of molecular states can be estimated from the number density of the molecular emitters, approximately 7 × 10^20^ cm^−3^, which, for a thickness of 70 nm, corresponds to ρ_mol_ = 4.9 × 10^7^ μm^−2^. Therefore, we obtain $N_{\text{eff}}=\frac{\rho_{\text{mol}}}{\rho_{\text{ph}}}=4\times 10^{6}$.

## Supplementary Material

http://advances.sciencemag.org/cgi/content/full/5/12/eaax4482/DC1

Download PDF

Inverting singlet and triplet excited states using strong light-matter coupling

## SUPPLEMENTARY MATERIALS

Supplementary material for this article is available at http://advances.sciencemag.org/cgi/content/full/5/12/eaax4482/DC1 Table S1. Hopfield model parameter fits. Fig. S1. Transient delayed PL in air and under vacuum. Fig. S2. Angle-resolved PL. Fig. S3. Transient delayed PL with different laser fluences. Fig. S4. Transient prompt PL characteristics.
