# Supplementary material for: Inverting singlet and triplet excited states using strong light-matter coupling
Source: Sci Adv. 2019 Dec 6;5(12):eaax4482. doi: 10.1126/sciadv.aax4482 (PMC6897552; doi:10.1126/sciadv.aax4482)
Supplement: http://advances.sciencemag.org/cgi/content/full/5/12/eaax4482/DC1 [file supp_5_12_eaax4482__index.html]

Science Advances | Science AdvancesAAASSearchScience AdvancesMenu

## Supplementary Materials

**This PDF file includes:**

- Table S1. Hopfield model parameter fits.
- Fig. S1. Transient delayed PL in air and under vacuum.
- Fig. S2. Angle-resolved PL.
- Fig. S3. Transient delayed PL with different laser fluences.
- Fig. S4. Transient prompt PL characteristics.

Download PDF

**Files in this Data Supplement:**

- Adobe PDF - aax4482\_SM.pdf
