# Supplementary material for: Inverting singlet and triplet excited states using strong light-matter coupling
Source: Sci Adv. 2019 Dec 6;5(12):eaax4482. doi: 10.1126/sciadv.aax4482 (PMC6897552; doi:10.1126/sciadv.aax4482)
Supplement: Download PDF [file aax4482_SM.pdf]

Science Advances
AAAS

advances.sciencemag.org/cgi/content/full/5/12/eaax4482/DC1

Supplementary Materials for

Inverting singlet and triplet excited states using strong light-matter coupling

Elad Eizner*, Luis A. Martínez-Martínez, Joel Yuen-Zhou, Stéphane Kéna-Cohen*

*Corresponding author. Email: eizner@polymtl.ca (E.E.); s.kena-cohen@polymtl.ca (S.K.-C.)

Published 6 December 2019, *Sci. Adv.* **5**, eaax4482 (2019)
DOI: 10.1126/sciadv.aax4482

This PDF file includes:

Table S1. Hopfield model parameter fits.
Fig. S1. Transient delayed PL in air and under vacuum.
Fig. S2. Angle-resolved PL.
Fig. S3. Transient delayed PL with different laser fluences.
Fig. S4. Transient prompt PL characteristics.

Table S1. Hopfield model parameter fits. Parameters obtained from a least square fit of the reflectivity data to the Hopfield Hamiltonian.

Sample	Polarization	$\Omega\ [eV]$	n_{eff}	$E_c\ [eV]$
MC 1	TE	0.45 ± 0.02	2.08 ± 0.11	2.54 ± 0.02
MC 1	TM	0.40 ± 0.01	3.50 ± 0.22	2.54 ± 0.02
MC 2	TE	0.50 ± 0.02	2.04 ± 0.10	2.41 ± 0.03
MC 2	TM	0.42 ± 0.02	3.26 ± 0.49	2.41 ± 0.03
MC Neat	TE	0.87 ± 0.01	1.97 ± 0.06	2.32 ± 0.02
MC Neat	TM	0.87 ± 0.02	2.86 ± 0.39	2.32 ± 0.02

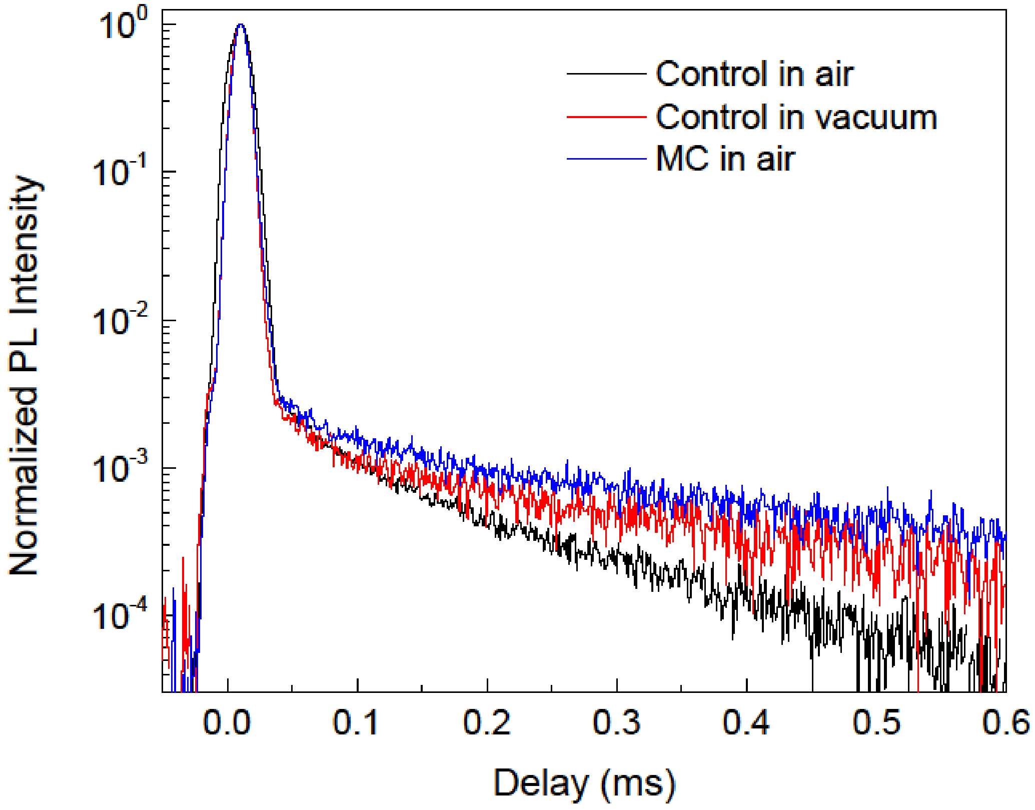

Fig. S1. Transient delayed PL in air and under vacuum. Transient PL decays for microcavity and control samples. Microcavity structure consisting of Al bottom mirror (75 nm), Al top mirror (25 nm), mCP buffer layers (10 nm each) and a 85 nm TADF layer of co-deposited mCP-3DPA3CN film (50% by volume).

–

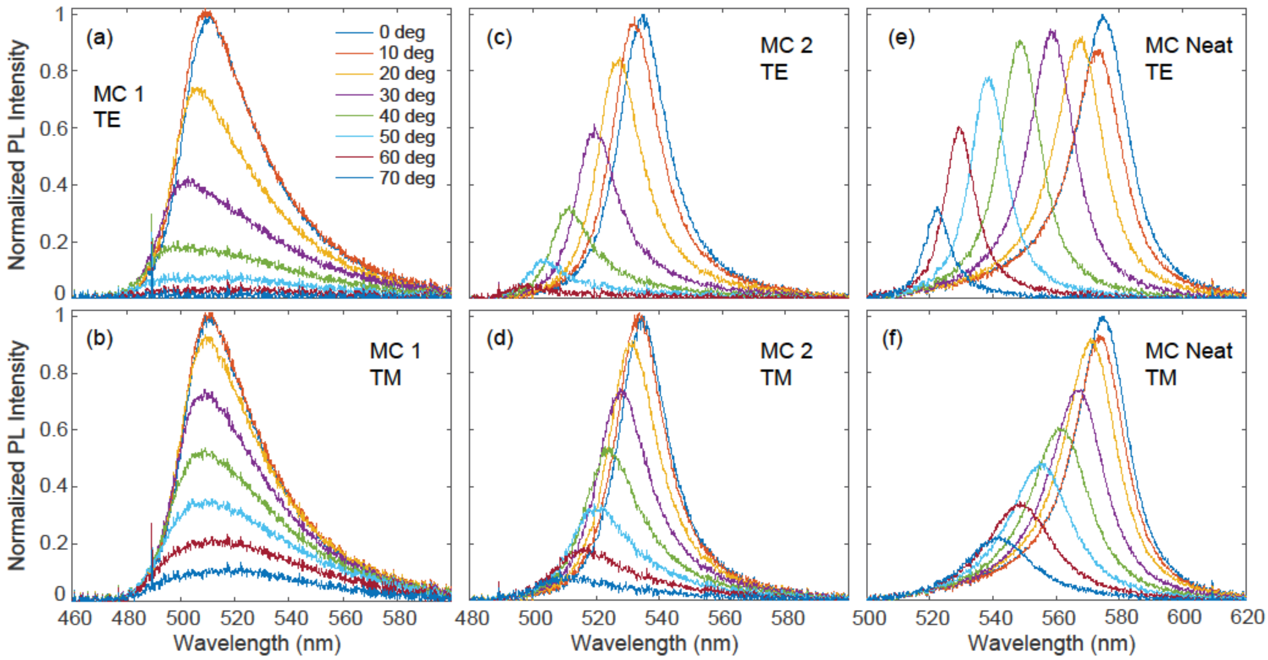

Fig. S2. Angle-resolved PL. For MC 1 ($\Omega = 0.45$ eV, $\Delta = -0.36$ eV) at (a) TE and (b) TM polarization, for MC 2 ($\Omega = 0.5$ eV, $\Delta = -0.49$ eV) at (c) TE and (d) TM polarization, and for MC Neat ($\Omega = 0.87$ eV, $\Delta = -0.58$ eV) at (e) TE and (f) TM polarization.

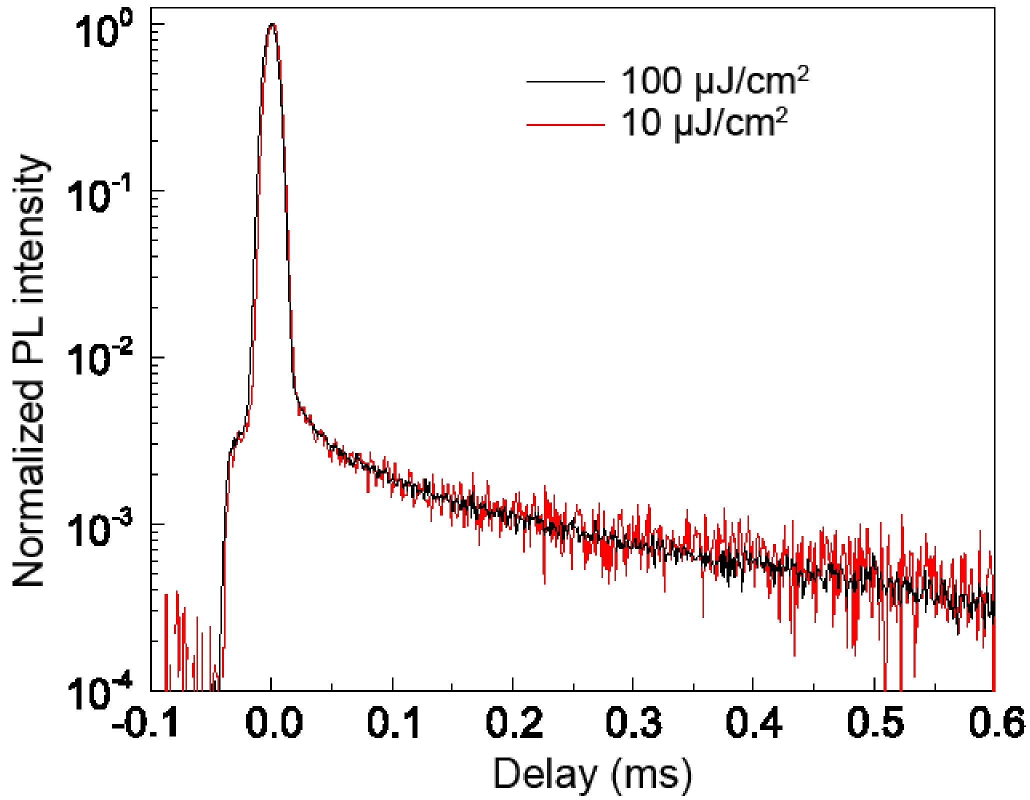

Fig. S3. Transient delayed PL with different laser fluences. Transient PL decays for co-deposited TPBi-3DPA3CN film (55% − 45% by volume) at excitation laser fluences of either 10 or 100 $\mu J/cm^2$. In this fluence range, no lifetime changes were found also for the microcavities.

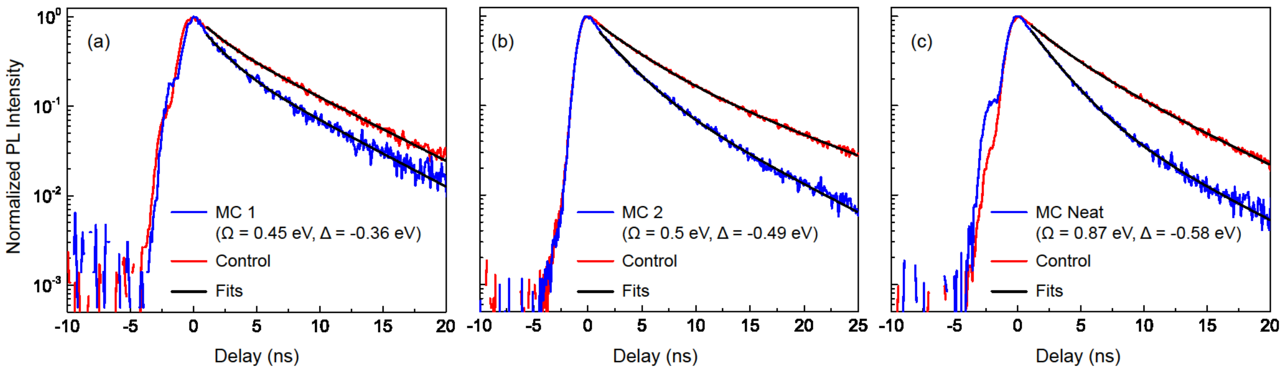

Fig. S4. Transient prompt PL characteristics. PL decays for the LP (blue line) and control film (red line). (a) MC 1, (b) MC 2 and (c) MC Neat. The black lines are multi-exponential fits to the prompt signal decay.
